# Comparison of T7E1 and Surveyor Mismatch Cleavage Assays to Detect Mutations Triggered by Engineered Nucleases

**DOI:** 10.1534/g3.114.015834

**Published:** 2015-01-07

**Authors:** Léna Vouillot, Aurore Thélie, Nicolas Pollet

**Affiliations:** Institute of Systems and Synthetic Biology, CNRS, Université d’Evry Val d’Essonne, Evry, France

**Keywords:** CEL nucleases, enzyme mismatch cleavage, mutation screening, T7 endonuclease I, *Xenopus*

## Abstract

Genome editing using engineered nucleases is used for targeted mutagenesis. But because genome editing does not target all loci with similar efficiencies, the mutation hit-rate at a given locus needs to be evaluated. The analysis of mutants obtained using engineered nucleases requires specific methods for mutation detection, and the enzyme mismatch cleavage method is used commonly for this purpose. This method uses enzymes that cleave heteroduplex DNA at mismatches and extrahelical loops formed by single or multiple nucleotides. Bacteriophage resolvases and single-stranded nucleases are used commonly in the assay but have not been compared side-by-side on mutations obtained by engineered nucleases. We present the first comparison of the sensitivity of T7E1 and Surveyor EMC assays on deletions and point mutations obtained by zinc finger nuclease targeting in frog embryos. We report the mutation detection limits and efficiencies of T7E1 and Surveyor. In addition, we find that T7E1 outperforms the Surveyor nuclease in terms of sensitivity with deletion substrates, whereas Surveyor is better for detecting single nucleotide changes. We conclude that T7E1 is the preferred enzyme to scan mutations triggered by engineered nucleases.

Genome editing using engineered nucleases such as zinc finger nucleases (ZFNs), transcription activator-like effector nucleases, and clustered regularly interspersed palindromic repeats (CRISPR)/CRISPR-associated protein 9 riboendonucleases is of considerable importance to contemporary molecular genetics. One prominent application concerns targeted mutagenesis. Once delivered to the cells, engineered nucleases can induce the cleavage of their target DNA sequence. This cleavage triggers cellular mechanisms of DNA repair that eventually will lead to mutations (Gaj *et al.* 2013; Terns and Terns 2014). These mutations are mostly deletions, but other mutations can be found, including insertions and insertions combined with deletions (Kim *et al.* 2013). Mutation hit-rate at a given locus needs to be evaluated because genome editing cannot target all loci with similar efficiencies. Moreover, in whole multicellular organisms, the delivery of engineered nucleases in one-cell stage embryos generally will lead to mosaic individuals. The genotype of such mosaic individuals can contain several mutant alleles with different frequencies and each mutation can be homo- or heterozygous at the cellular level. Thus, the subsequent analysis of mutant organisms obtained using engineered nucleases requires powerful techniques for large-scale mutation detection. Such techniques should allow the detection of one mutated allele in a background of wild-type (WT) alleles. Moreover, they should be reproducible, reliable, inexpensive, and should deliver clear results for all kinds of unknown mutations. These techniques are needed at the screening stage, upstream of the sequencing process that will ultimately identify the mutant DNA sequence. Yet, most mutation detection methods are not applicable to this field because they do not generally achieve high selectivity, they need specific sequence context, they are laborious, they need specialized instrumentation, or they are not amenable to routine analysis (Yeung *et al.* 2005).

Today, we can quantify mutation hit-rate after the delivery of engineered nucleases by sequencing a representative panel of cloned polymerase chain reaction (PCR) products obtained from different individuals. The sequencing is done either by the traditional Sanger method or by next-generation sequencing, both of which being rather expensive approaches requiring several days to complete. High-resolution melting curve analysis recently has been proposed to identify mutations, but this method requires the development of a specialized quantitative PCR assay on small PCR products (Dahlem *et al.* 2012).

Another strategy to identify unknown mutations relies on the identification of heteroduplex DNA formed after melting and hybridizing mutant and WT alleles. The identification of heteroduplex DNA can be done with chemicals, enzymes, or proteins that bind mismatches (Dodgson and Wells 1977; Bhattacharyya and Lilley 1989; Wagner *et al.* 1995; Youil *et al.* 1995; Howard *et al.* 1999; Taylor and Deeble 1999; Yeung *et al.* 2005). The enzyme mismatch cleavage (EMC) method takes advantages of enzymes able to cleave heteroduplex DNA at mismatches formed by single or multiple nucleotides. It is known that the cleavage activity of such enzymes is greater on mismatched than on Watson-Crick base pairs, but homoduplex DNA can be cleaved to a certain extent (Babon *et al.* 1995; Youil *et al.* 1995).

The first enzymes used for EMC were bacteriophage resolvases such as T4E7 and T7E1 (Youil *et al.* 1995; Mashal *et al.* 1995). The structure, function, and substrate specificity of T7E1 has been well studied (Hadden *et al.* 2007; Freeman *et al.* 2013). The substrates of T7E1 are distorted dsDNA undergoing conformational changes (Déclais and Lilley 2008). Thus, T7E1 can recognize and cleave different dsDNA molecules if their structure is kinked and able to bend further (Mashal *et al.* 1995; Déclais *et al.* 2006). Typically, heteroduplex dsDNA containing bulges formed by extra-helical loops and even single base mismatches can adopt such polymorphic structures (Gohlke *et al.* 1994). Conversely, perfectly paired homoduplex dsDNA will not constitute good substrates for T7E1. The ability of T7E1 to discriminate between homoduplex and heteroduplex dsDNA has formed the basis of a mutation detection assay that worked with moderate success (Mashal *et al.* 1995). Yet the sequence and the number of mismatched nucleotides and the flanking sequence between the two DNA strands affect the heteroduplex structure and will therefore affect the cleavage efficiency by T7E1. This is why deletions are cleaved more efficiently than single base mutations as noted by Mashal *et al.* (1995).

Plant single-strand specific endonucleases of the S1 nuclease family such as CEL and ENDO have been used more recently for mutation detection (Oleykowski *et al.* 1998; Yang *et al.* 2000; Qiu *et al.* 2004; Triques *et al.* 2008, 2007). CELI, CELII (commercialized under the brand Surveyor), and ENDO1 are single-stranded nucleases active on DNA or RNA. These nucleases do not form dimers and they cleave DNA only one strand at a time on the 3′ side of a mismatch (Oleykowski *et al.* 1998; Yang *et al.* 2000; Qiu *et al.* 2004; Yeung *et al.* 2005; Voskarides and Deltas 2009). They have been described as useful for EMC assays but they contain also 5′ exonuclease activity (Qiu *et al.* 2004; Till *et al.* 2004). The specificity of CEL nucleases to identify single-base mismatches has been extensively and empirically proven by its use in the reverse genetics strategy of TILLING (Bentley *et al.* 2000; Colbert *et al.* 2001; Coghill *et al.* 2002; Perry *et al.* 2003; Wienholds *et al.* 2003; Comai *et al.* 2004; Till *et al.* 2004; Slade *et al.* 2005). Yet there is limited evidence of their activity on extrahelical loops. The initial reports have shown an ability to detect extrahelical loops of up to 11 or 12 nucleotides (Oleykowski *et al.* 1998; Qiu *et al.* 2004). Despite the preference of CEL and ENDO in cleaving single nucleotide mismatches, the Surveyor-based EMC assay is used commonly to scan mutations induced by engineered nucleases (Beumer *et al.* 2008; Geurts *et al.* 2009; Guschin *et al.* 2010; Miller *et al.* 2011; Tesson *et al.* 2011; Isalan 2012; Sanjana *et al.* 2012; Holkers *et al.* 2013; Maier *et al.* 2013; Van Rensburg *et al.* 2013).

EMC assays are cost-effective methods that can be performed with the use of simple laboratory setups. This is why T7E1 and Surveyor Mismatch nucleases enzymes are used widely in the context of engineered nucleases mutagenesis projects. Yet, the spectrum of mutations that can be induced by engineered nucleases is broad (Kim *et al.* 2013). The choice of one or the other EMC enzyme, however, is currently lacking a side-by-side comparison. Different teams compared these enzymes in the past but not in the framework of mutation induced by ZFNs, transcription activator-like effector nucleases, or CRISPR/CRISPR-associated protein 9 riboendonucleases (Tsuji and Niida 2008).

We present here the first comparison of the sensitivity of T7E1 and Surveyor EMC assays on deletions and point mutations obtained by ZFNs in frog embryos. We report here our findings on the mutation detection limits and efficiency of T7E1 and Surveyor.

## Materials and Methods

### Construction of WT and mutant plasmids

We used the *Xenopus tropicalis smn2* gene and its exon-intron structure as previously described as a starting point to design our plasmid constructs (GenBank NM_001100240) (Ymlahi-Ouazzani *et al.* 2010). We targeted three genomic segments containing exons 2a (488 bp, 35.0% GC), exon 3 (572 bp, 37.6% GC), and exon 6 (500 bp, 37.4% GC) of *smn2* (Supporting Information, Figure S1). We amplified these three segments from 100 ng of genomic DNA (*X. tropicalis* TGA strain) using primers pairs described in Table S1. We cloned these PCR products, and all the others described herein, into pCRII Vector using TA Cloning Kit (Life Technologies) and checked them by sequencing. We then used these constructs as the WT PCR products. We used an overlap extension PCR approach to produce 20-bp deletions in these three genomic segments (Figure S1 and Table S1) (Ho *et al.* 1989). We designed the position of the deletion so that a cleavage would lead to two products with significant size differences (150−200 bp). We cloned the three deletion mutant DNA segments and checked them by sequencing. We obtained the D15 and D19 alleles from experiments of genome engineering using ZFNs. In short, we microinjected ZFN RNAs targeting *smn* exon2a sequences into one-cell-stage *X. tropicalis* embryos. We extracted genomic DNA from pool of ten tailbud-stage embryos (Nucleospin tissue kit; Macherey-Nagel). We amplified the segments containing the exon2a by PCR from 100 ng of genomic DNA with the aforementioned primers. We purified the PCR products and cloned them. We checked 10 clones by sequencing and selected the mutant clones D15 and D19.

### PCR

We used 1 ng of plasmidic DNA template in our standard PCRs. Each 20-µL PCR contained 10 µL of One*Taq* Hot Start 2X Master Mix (New England Biolabs), 400 nM of each primer and nuclease-free water. PCR conditions were 1 cycle of 5 min at 95°, followed by 40 cycles of 30 sec at 95°, 30 sec at 50° or 58° (depending on the primer pairs, see Table S1), and 30 sec at 72°, and finishing with 5 min incubation at 72°. We checked PCR products amplification and concentration by spectrophotometry and standard gel electrophoresis.

### Heteroduplex formation

We mixed 250 ng of PCR products obtained from WT and mutant plasmids and denatured them by heating at 99° for 5 min in a thermocycler (Biometra). We then formed the heteroduplexes by cooling down to 65° for 30 min and to 23° for 30 min using a thermocycler.

### Digestion by T7 endonuclease I or Surveyor

We performed T7 endonuclease I digestion at 37° for 30 min using 5 U of T7 endonuclease I (T7E1; New England BioLabs) on 20 µL of unpurified PCR products (~250 ng) in a reaction volume of 50 µL. We stopped the reaction by adding 4 µL of 0.5 M ethylenediaminetetraacetic acid. We performed the Surveyor nuclease assay using 20 µL of unpurified PCR products incubated with 1 µL of Surveyor nuclease (Transgenomic SURVEYOR mutation detection kit for standard gel electrophoresis), 1 µL of Surveyor Enhancer, and 4 µL of 0.15 M MgCl_2_ in a 50-µL reaction. We incubated the mix for 1 hr at 42° and stopped the reaction by adding 4 µL of the stop solution provided in the kit. The reactions were either kept at −20° or used immediately for electrophoresis.

### Electrophoresis

To assess T7E1 and Surveyor nuclease digestion, we performed capillary electrophoresis on an Agilent 2100 Bioanalyzer. We used the DNA 1000 LabChip kit for electrophoresis of double-stranded DNA with detection by an intercalating dye and loaded 1 µL of the unpurified reaction products on the DNA chips. We used the 2100 Expert software (Agilent Technologies) for quantification and sizing of the digestion products. Data were exported in .csv files and electropherograms were drawn using R statistical software scripts.

## Results

We first compared the efficiency of two enzymatic assays based on mismatch cleavage: T7E1 and Surveyor. We set up an assay in which we mixed different ratios of WT and deletion mutant DNA to produce heteroduplex molecules. We analyzed the cleavage products by using capillary lab-on-a-chip nondenaturing electrophoresis on a Bioanalyzer. We reasoned that such assays could be dependent on the PCR product sequence and thus we analyzed three different PCR products. The sequences of the three PCR products are different, and their GC content ranged from 35.0 to 37.6%, close to the genome wide GC content (40.1%). Because we know that most mutations induced by engineered nucleases are deletions, we constructed 20-bp deleted versions of each PCR product (Figure S1). We will refer to these three sets of different PCR products as *smn* exon 2a, *smn* exon 3, and *smn* exon 6.

**Figure 1:**
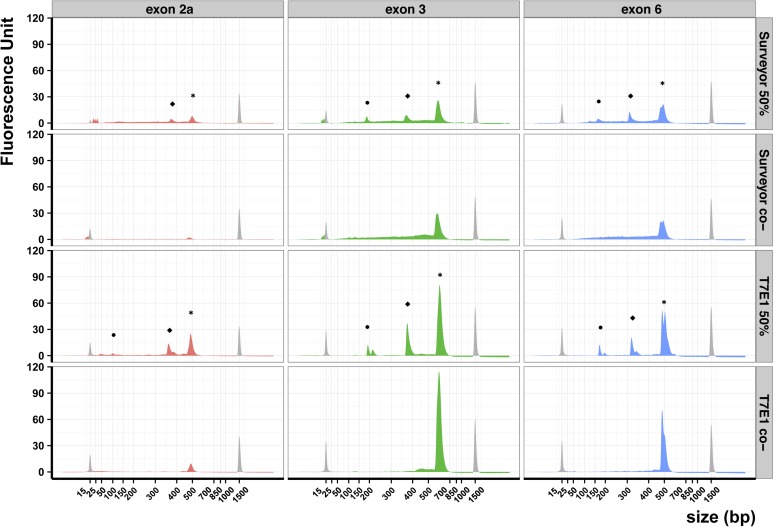
Comparison of Surveyor and T7E1 efficiencies. Electropherograms obtained on three different DNA products (exon 2a, exon 3, and exon 6) treated using either the Surveyor (top two rows) or T7E1 enzyme (bottom two rows). The Surveyor and T7E1 assays were made on a 50% mix of wild-type and 20-bp deletion mutants. For the Surveyor and T7E1 negative controls (co-), we prepared individual reactions using only wild-type or mutant DNA and pooled the products before electrophoresis. Peaks colored in gray correspond to the internal molecular weight standards: the small molecular weight marker measures 15 bp and the large measures 1500 bp. Peaks labeled with an asterisk (*) correspond to uncleaved DNA, peaks labeled • and ⬥ correspond to cleaved DNA heteroduplex.

In a first set of experiments, we mixed WT and mutant PCR products in equal quantities before heteroduplex formation. We expected that treatment with T7E1 or Surveyor nucleases would lead to cleavage of heteroduplex molecules in two smaller molecules. Besides these cleavage products, we expected uncleaved WT or mutant homoduplexes, and eventually some uncleaved heteroduplexes. These uncleaved DNA would altogether compose the main peak (peaks *, Figure 1) on an electropherogram provided by a BioAnalyzer fragment analysis. By design, we know that the WT and mutant homoduplexes differ in size by 20 bp but our electrophoresis assay cannot distinguish these products for exon 2a and exon 3, whereas it can for exon 6 (peaks *, Figure 1). The smaller molecules generated by cleavage would compose the two smaller peaks on an electropherogram (peaks • and ⬥, Figure 1). Thus, we would expect the nuclease digestion products would give rise to three peaks on an electropherogram.

When equal quantities of mutant and WT DNA were used, we observed the same number of peaks with both nucleases: two peaks were distinguishable for exon 2a and three for exons 3 and exons 6 (Figure 1). The sizes of the generated fragments corresponded to what we expected from the deletion’s site. In all three cases the peak fluorescence values obtained with Surveyor were lower than those obtained with T7E1 (Figure 1). This explained why the smallest fragment (peak •) was not observed for exon 2a using Surveyor. Furthermore, the baseline of the electropherogram was neither straight nor regular in all samples treated by Surveyor because of exonucleolytic activity, as previously reported (Qiu *et al.* 2004; Till *et al.* 2004). This was particularly striking in the control samples for exon 3 and exon 6. In addition small peaks located near the 15-bp molecular weight marker in all samples treated by Surveyor suggested an aspecific digestion of the PCR products. Because we used the same quantity of DNA in all reactions, these lower fluorescence values and irregular base line indicated a loss of DNA due to the Surveyor treatment. Both T7E1 and Surveyor were able to cleave heteroduplexes independently of the PCR product. Yet, T7E1 treatment leads to a higher signal to noise ratio than the Surveyor treatment.

**Figure 2:**
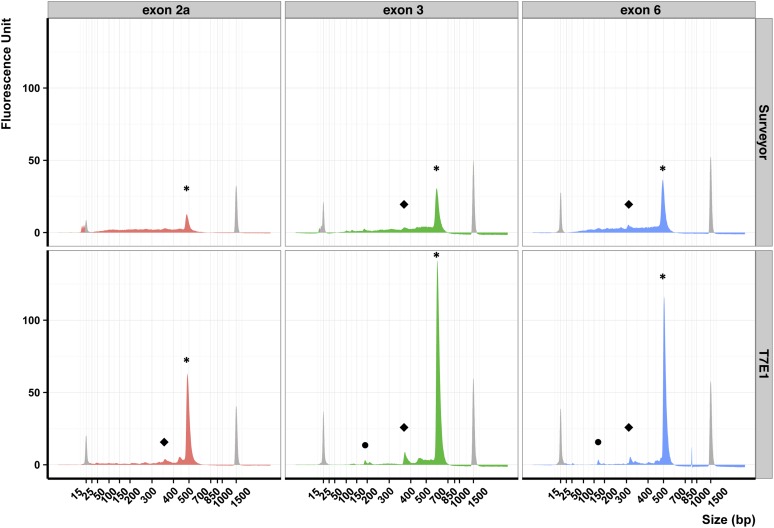
Limits of sensitivity of Surveyor and T7E1 on different templates. Electropherograms obtained on DNA products from exon 2a, 3, and 6 after Surveyor or T7E1 digestion. The Surveyor and T7E1 assays were made on a mix composed of 5% 20-bp deletion mutants and 95% of wild-type DNA. Peaks colored in gray correspond to the internal molecular weight standards: the small molecular weight marker measures 15 bp and the large measures 1500 bp. Peaks labeled with an asterisk (*) correspond to uncleaved DNA, peaks labeled • and ⬥ correspond to cleaved DNA heteroduplex.

We then evaluated the sensitivity of T7E1 and Surveyor for our three different DNA products. When we mixed 5% of mutant DNA with WT DNA, we observed only one peak for exon 2a after Surveyor treatment and two or three peaks for all other cases (Figure 2). We observed the largest peak obtained from the cleavage of heteroduplexes in all three DNAs samples treated by T7E1 (Figure 2, peaks ⬥). We could guess this same peak by using the Surveyor assay on exon 3 DNA sample, but we could not observe this peak using the Surveyor assay on exon 2a DNA sample. In addition, we could never observe the smallest peak in DNA samples treated by Surveyor (Figure 2, peaks •). The T7E1 assay results were always easier to interpret than the Surveyor. In conclusion, these results showed that the T7E1 assay enables the detection of 5% of heteroduplex DNA in a background of WT DNA.

**Figure 3:**
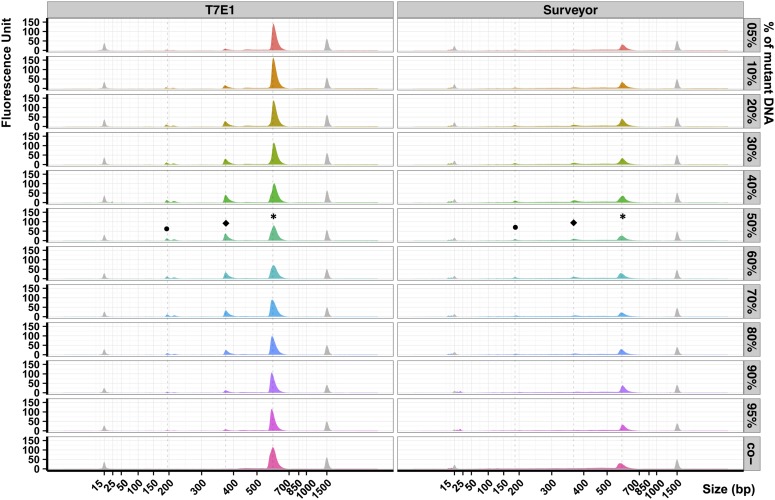
Comparison of Surveyor and T7E1 sensitivity. Electropherograms obtained on exon 3 DNA products after Surveyor or T7E1 digestion. The percentage indicated on the right corresponds to the quantity of mutant DNA in a pool of mutant and wild-type DNA. The Surveyor and T7E1 negative controls (co-) correspond to reactions made using mutant DNA only (100%). Peaks colored in gray correspond to the internal molecular weight standards: the small molecular weight marker measures 15 bp and the large measures 1500 bp. Peaks labeled with an asterisk (*) correspond to uncleaved DNA, peaks labeled • and ⬥ correspond to cleaved DNA heteroduplex.

To detect the limits of sensitivity for T7E1 and Surveyor assay, we made mixes of mutant and WT exon 3 DNAs in different ratios from 5% up to 100% of mutant DNA. When digested by T7E1, the intensity of the main peak (*) was inversely proportional to the homoduplex:heteroduplex ratio (Figure 3, peak *). Conversely, the intensities of the smaller peaks were proportional to the homoduplex/heteroduplex ratio. We detected cleavage products even for 5% of heteroduplex DNA (Figure 3, peaks • and ⬥, samples 5% and 95%). We could see that the smallest peak was a doublet that differed by 20 bp. This 20-bp size difference corresponds to the size of the deletion. On the basis of these results, we conclude that the T7E1 assay could detect around 5% of mutant DNA.

When we digested the DNAs with Surveyor, we did not observe the same sensitivity pattern. We detected the main peak, but its value of fluorescence was three times lower than with T7E1, even though we used the same quantity of starting DNA. In line with this observation, we observed small peaks around the 15-bp DNA marker. Besides, all Surveyor treated samples led to greater electropherogram base lines in comparison with T7E1-treated ones. These results suggested again an exonucleolytic activity as previously reported. Yet we could detect from 10 to 90% of mutant DNA using Surveyor (Figure 3).

**Figure 4:**
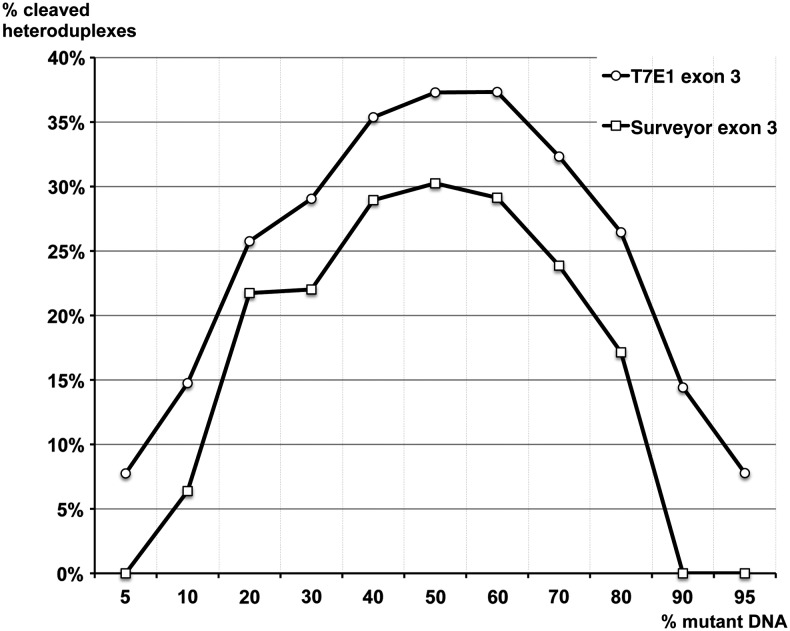
Quantification of Surveyor and T7E1 efficiency and sensitivity. This graph shows the fraction of cleaved products from all products (y-axis) in a mixture composed of various quantities of deletion mutants of exon 3 in a population of mutant and wild-type DNA molecules (x-axis). Open circles correspond to T7E1 digestion products, open squares correspond to Surveyor digestion products.

We then quantified the efficiency of these two enzymes by comparing the quantity of cleaved PCR products (Figure 4 and Figure S2). In theory, when mutant and WT DNA are present in equal quantities, we can form up to 50% of heteroduplex DNA molecules. We quantified the quantity of cleaved heteroduplexes and we expressed our results as percentages of cleaved heteroduplexes relative to the total quantity of DNA.

We observed a symmetry on either side of 50% of mutant DNA that correlates with the results presented in Figure 3. T7E1 cleaved nearly all heteroduplexes for mixes containing 30 and 70% of mutant DNA. Surveyor cleaved nearly all heteroduplexes for mixes containing 20 and 80% of mutant DNA. We did not observe any Surveyor cleavage product for 5, 90, and 95%. The quantity of cleaved heteroduplexes with T7E1 was always 8% superior to that obtained with Surveyor. In the range of 40–60%, the quantity of cleaved product reached a plateau: 30% for Surveyor (*i.e.*, 60% of all heteroduplexes) and 37% for T7E1 (*i.e.*, 74% of all heteroduplexes).

**Figure 5:**
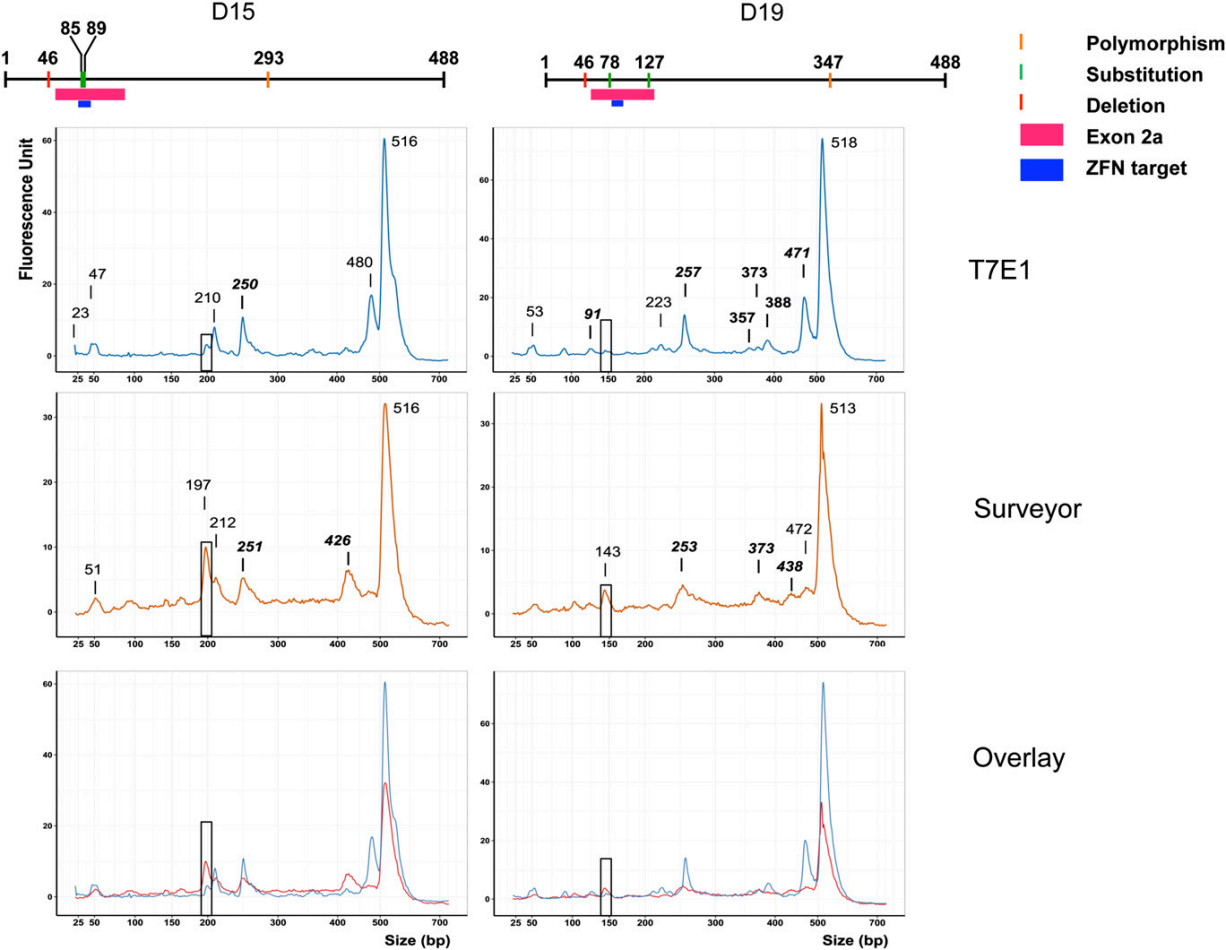
Comparison of Surveyor and T7E1 efficiencies on point mutations. The top of this figure presents schematics of the D15 and D19 sequence differences in comparison with the wild-type *smn* exon 2a. The electrophoregrams obtained on D15 or D19 DNA products are presented from top to bottom in the following order: after T7E1 digestion, after Surveyor digestion, and an overlay of the two conditions. The size of the peaks is given in base pairs. Only peaks called by the software were labeled. Peaks matching the size of a partial digestion product are written in bold italics. Boxed peaks are discussed in the text. The portions of the small and large markers have been omitted for clarity.

We then tested these enzymes on two mutant DNA molecules obtained from ZFN-injected *Xenopus* embryos. The sequencing data obtained for clones D15 and D19 showed that both alleles contained a single-nucleotide deletion polymorphism at position 46 of the exon 2a amplicon. The D15 allele contained two single nucleotide substitutions (A > G) at position 85 and 89 of the amplicon (position 29 and 33 of *smn* exon 2a) and one SNP at position 293 (T > A). The second allele, D19, contained two single-nucleotide substitutions (T > C) at position 78 and 127 of the amplicon (position 22 and 71 of *smn* exon 2a) and one SNP at position 347 (T > C). To test the Surveyor and T7E1 assay on these complex substrates, we set up a digestion reaction composed of a mix containing 50% of the mutant alleles and 50% of the WT allele. We expected five DNA fragments from a whole digest for D15 (4, 39, 46, 195, and 204 bp) and D19 (32, 46, 49, 141, and 220 bp) heteroduplexes (Figure S3 and Figure S4). The 5-bp digestion product for D15 could not be visualized. After T7E1 or Surveyor digestion, we observed a complex pattern of cleavage products (Figure 5). We reasoned that the digestions could be partial, so that more DNA fragments would be produced. Indeed, most peaks distinguishable on the electropherogram could fit a DNA fragment produced by an incomplete digestion (Table S2). The most visible example can be seen on Figure 5 as peaks of about 400 bp resulting from a single cleavage at position 46 of the amplicon. Yet, we noticed that the single-nucleotide polymorphisms (*i.e.*, SNPs) SNP 293 from D15 and SNP 347 from D19 were cleaved with better efficiency when Surveyor nuclease was used (boxed peaks in Figure 5). For D15, we observed a peak of 197 bp reaching about 10 fluorescence units (FUs) with Surveyor, whereas a peak at 200 bp only reached about 3 fluorescence units (FUs) and was not even marked for T7E1 (Figure 5). Similarly for D19, the 141-bp fragment resulting from cleavage of SNP 347 was digested more efficiently with Surveyor (peak at 143 bp), but we could not see a signal above the background at this size with T7E1. In conclusion, it appears that Surveyor outperforms T7E1 for single-nucleotide changes.

## Discussion

We showed that both T7E1 and Surveyor enabled the detection of a mutant allele present in half of a DNA mixture (Figure 1). Thus, both assays can work on heterozygous individuals, and Surveyor was able to cleave heteroduplex substrates containing a 20 nucleotides bulge. As expected from substrate preferences, the signal-to-noise ratio is clearly greater for T7E1 than for Surveyor. Similar results have been reported using a regular agarose gel electrophoresis assay (Sakurai *et al.* 2014). The known 5′-exonuclease activity of Surveyor is probably responsible of DNA degradation resulting in greater background. The exonuclease activity of Surveyor has previously been reported (Till *et al.* 2004; Yeung *et al.* 2005) and shown to be highly dependent on the concentration of Mg^2+^ and thus on the purification method and on the PCR buffer. We added MgCl_2_ to the reaction to avoid this effect as much as possible. Since we obtained similar results on purified and unpurified PCR products (data not shown) we opted for the simpler and economical solution to avoid the PCR purification step.

We could detect a 20-bp deletion mutation in a pool of DNA containing as little as 5% of a mutant allele when we used T7E1. When we used the Surveyor assay, the detection limit was closer to 20%. The exonuclease activity of Surveyor produced too many small DNA fragments that masked a potentially specific signal. For example we observed accumulation of DNA fragments below 25 bp (Figure 2 and Figure 3). Using T7E1, when the cleavage products are sufficiently small, we can detect pairs of cleavage products separated by 20 bp. This size difference arise from T7E1 cleaving on the 3′ end of the mismatches, thereby producing two fragments including one with a 20 bp overhang. Thus the size difference very likely corresponds to the size of the deletion in our mutant allele.

The two enzymes exhibited different behaviors. T7E1 showed a maximum efficiency in a DNA pool containing between 5 and 30% of 20-bp deletion mutant DNA, and by symmetry from 70 to 95% of mutant DNA (Figure 4). A plateau was reached when the heteroduplex proportion was maximal, *i.e.*, at 50% of mutant DNA in the pool. We observed that at best T7E1 cleaved about 80% of heteroduplexes in the DNA pool.

Because of the high background of nonspecific cleavage products by Surveyor assay, we could not reliably evaluate its efficiency on a DNA pool containing from 5 to 30% of mutant molecules. It is possible that the enzyme cut all the heteroduplex at the desired location, but it is possible that the 5′ exonuclease activity of Surveyor further process the cleaved products to generate size heterogeneity. Thus the observed Surveyor specificity was much lower than that of T7E1 in our protocol. We observed that at best Surveyor cleaved 60% of heteroduplexes DNA, a lower efficiency than T7E1.

The different activities of these enzymes according to the precise nature of the mismatch were already known (Tsuji and Niida 2008). T7E1 identifies preferentially insertions and deletions, whereas Surveyor is better to recognize substitutions. Here we showed that the surrounding DNA sequence did not impact their activity (Figure 1 and Figure 2). On the three DNA fragments tested, we could measure the limit of sensitivity for Surveyor (10% of mutant DNA) and for T7E1 (5%). Thus, T7E1 was always more sensitive than Surveyor in our assays using deletion heteroduplex substrates.

We report here results obtained using a simple, reproducible, and economic protocol. Any molecular biology laboratory can use this protocol as a first step for mutation screening before sequencing. The whole procedure starting from genomic DNA can be performed in a single day of work, in contrast with the recently reported application of HRM analysis that requires specific instrumentation and the development of a specific PCR assay (Dahlem *et al.* 2012). We could detect 5% of deletion mutant DNA using T7E1. Yet, the reported mutation hit rate in F0 individuals issued from nuclease injections ranges from 10 to 47% (Young *et al.* 2011; Ishibashi *et al.* 2012; Lei *et al.* 2012; Nakajima and Yaoita 2013; Suzuki *et al.* 2013; Guo *et al.* 2014). Thus, this T7E1 assay is sensitive enough to identify mutations in DNA samples obtained from F0 individuals. In addition a pooling strategy can be implemented to genotype F1 individuals. 5% sensitivity level corresponds roughly to one heterozygous mutant in a pool of eight individuals (one mutant allele of 16 alleles, *e.g.*, 6.25%). Using such a pooling strategy we could screen 96 individuals on a single Bioanalyzer DNA chip. Moreover, pooling can be beneficial for mutation detection, since the fraction of heteroduplex increases with the pool size.

One possible bias in the EMC approach comes from the production of the template DNA using PCR. If the fidelity of the DNA polymerase is limited, mutations can be introduced in the template. Even if the proportions are expected to be low, this can impact the EMC assay in an unpredictable way. A solution to this problem is to use proof-reading polymerases. Yet such proof-reading polymerases could introduce biases when the quantity of mutant molecules in the template DNA is low. Upon denaturation and hybridation cycles, and especially in the late PCR cycles, heteroduplex dsDNA are formed and could lead to an increase of recombined PCR products (Judo *et al.* 1998). It has been shown that proof-reading polymerases lead to increased levels of recombined PCR products (Judo *et al.* 1998; Lahr and Katz 2009). Such recombination events during PCR could lead to dsDNA in which the mutation to be detected is completely or partially corrected. Another problem introduced by PCR is allele drop-out, the amplification bias of one allele *vs.* another, in one way or another. In particular deletions can affect the melting temperature of the PCR product. Usually, this effect tends to promote the amplification of smaller PCR products. Thus, the production of the template DNA using PCR can affect the sensitivity of the EMC assay and the estimation of the original quantity of the mutant allele. It has been shown that recombination during PCR can be limited by reducing the quantity of starting DNA, reducing the number of PCR cycles, and increasing extension time (Judo *et al.* 1998; Lahr and Katz 2009). Similarly any genetic polymorphism will affect the usefulness of EMC to detect mutations, and it is crucial to examine the extent of polymorphisms on the DNA region amplified by PCR. The overall design of the PCR assay, including the amplicon size, is thus of paramount importance. Several studies took advantage of restriction enzymes sites to assess the mutation hit rate in given conditions. Yet it may be impossible to find a unique restriction site near the nuclease target sequence. Because the EMC assays can be used whatever the sequence of the PCR template, it is a more versatile strategy.

The EMC assay can benefit from several optimizations. For example, the use of fluorescent oligonucleotides could improve the signal-to-noise ratio, especially if small products are expected from the cleavage (Behrensdorf *et al.* 2002). The exonuclease activity of Surveyor leads to the nibbling of dsDNA and a lower signal-to-noise ratio. Using 5′ fluorescently labeled oligonucleotides has been shown to increase dramatically the sensitivity of CEL assays (Till *et al.* 2004). This effect has been explained by the combination of the protection provided by the 5′ label against 5′ exonuclease activity, and an increased signal to noise ration since degraded products lose the label (Till *et al.* 2004). The use of a ligase has also been reported to improve the results (Huang *et al.* 2012). However, these ameliorations are associated with a loss of simplicity and an increase in costs.

The two enzymes tested exhibit an exonuclease activity to some extent. Such a nonspecific exonuclease activity can be reduced by the combination of the EMC enzyme with ampligase (Huang *et al.* 2012). In their paper, Huang *et al.* (2012) discuss two important factors affecting the exonuclease activity of the EMC enzymes. The first factor is the buffer used for the enzymatic incubation, and the second factor is the incubation time. It is crucial to avoid the incubation in the PCR buffer because it counteracts the effects of the ampligase. The incubation time should not exceed 30 min, and should ideally be 20 min long. Yet, these conditions are completely dependent of the enzyme used. The authors worked with samples containing 10 or 50% of single base mutant DNA. Here, we showed that T7E1 enabled the detection of 5% deletion mutant DNA. The exonuclease activity of Surveyor without ampligase led to the appearance of many small DNA fragments that masked the specific signal coming from heteroduplex digestions. In conclusion, we showed that T7E1 outperforms the Surveyor nuclease in terms of sensitivity with deletion heteroduplex substrates and should be preferred here.

## Supplementary Material

Supporting Information
