# Supplementary material for: Comparison of T7E1 and Surveyor Mismatch Cleavage Assays to Detect Mutations Triggered by Engineered Nucleases
Source: G3 (Bethesda). 2015 Jan 7;5(3):407–15. doi: 10.1534/g3.114.015834 (PMC4349094; doi:10.1534/g3.114.015834)
Supplement: Supporting Information [file supp_5_3_407__index.html]

Comparison of T7E1 and Surveyor Mismatch Cleavage Assays to Detect Mutations Triggered by Engineered Nucleases — Supporting Information 

# Comparison of T7E1 and Surveyor Mismatch Cleavage Assays to Detect Mutations Triggered by Engineered Nucleases

## Supporting Information for Vouillot, Thélie, and Pollet, 2015

**Files in this Data Supplement:**

- Supporting Information - Figures S1-S4 and Tables S1-S2 (PDF, 334 KB)
- Figure S1 - Structure of the PCR products corresponding to *smn* exon 2a, exon 3 and exon 6. (PDF, 415 KB)
- Figure S2 - This graph shows the fraction of cleaved products from all products (y axis) in a mixture composed of various quantities of deletion mutants of exon 2a, 3 and 6 in a population of mutant and wild-type DNA molecules (x axis). (PDF, 336 KB)
- Figure S3 - Predicted cleavage map of all mismatches between D15 and wild-type *smn* exon 2a alleles. (PDF, 423 KB)
- Figure S4 - Predicted cleavage map of all mismatches between D19 and wild-type *smn* exon 2a alleles. (PDF, 425 KB)
- Table S1 - Sequence of primers used in this work. (PDF, 130 KB)
- Table S2 - List of fragments obtained after T7E1 or Surveyor assays on D15 and D19 clones. (PDF, 103 KB)
