# Supplementary material for: Comparison of T7E1 and Surveyor Mismatch Cleavage Assays to Detect Mutations Triggered by Engineered Nucleases
Source: G3 (Bethesda). 2015 Jan 7;5(3):407–15. doi: 10.1534/g3.114.015834 (PMC4349094; doi:10.1534/g3.114.015834)
Supplement: Supporting Information [file supp_g3.114.015834_015834SI.pdf]

G3 · Genes | Genomes | Genetics

Comparison of T7E1 and Surveyor mismatch cleavage assays to detect mutations triggered by engineered nucleases

Léna Vouillot, Aurore Thélie and Nicolas Pollet

Institute of Systems and Synthetic Biology, CNRS, Université d'Evry Val d'Essonne, Bâtiment 3, Genopole® campus 3, 1, rue Pierre Fontaine, F-91058 Evry, France.

Corresponding author: Nicolas Pollet, Institute of Systems and Synthetic Biology, CNRS, Université d'Evry-Val-d'Essonne, Genavenir 3, Genopole campus 3, 1, rue Pierre Fontaine, F-91058 Evry, France, Phone +33 (0)164982748 Fax. +33 (0)169361119 Email: Nicolas.Pollet@issb.genopole.fr

DOI: 10.1534/g3.114.015834

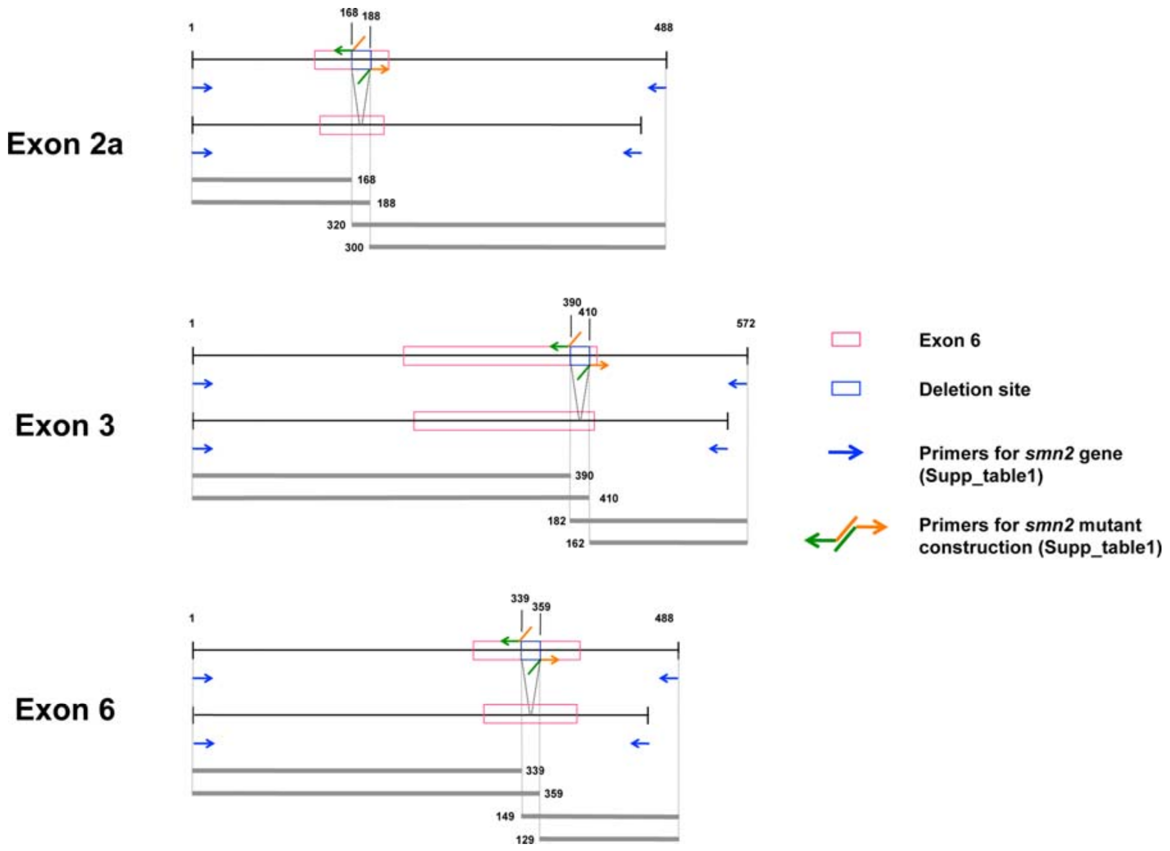

Figure S1 Structure of the PCR products corresponding to *smn* exon 2a, exon 3 and exon 6. Both the wild-type and constructed mutant forms are depicted.

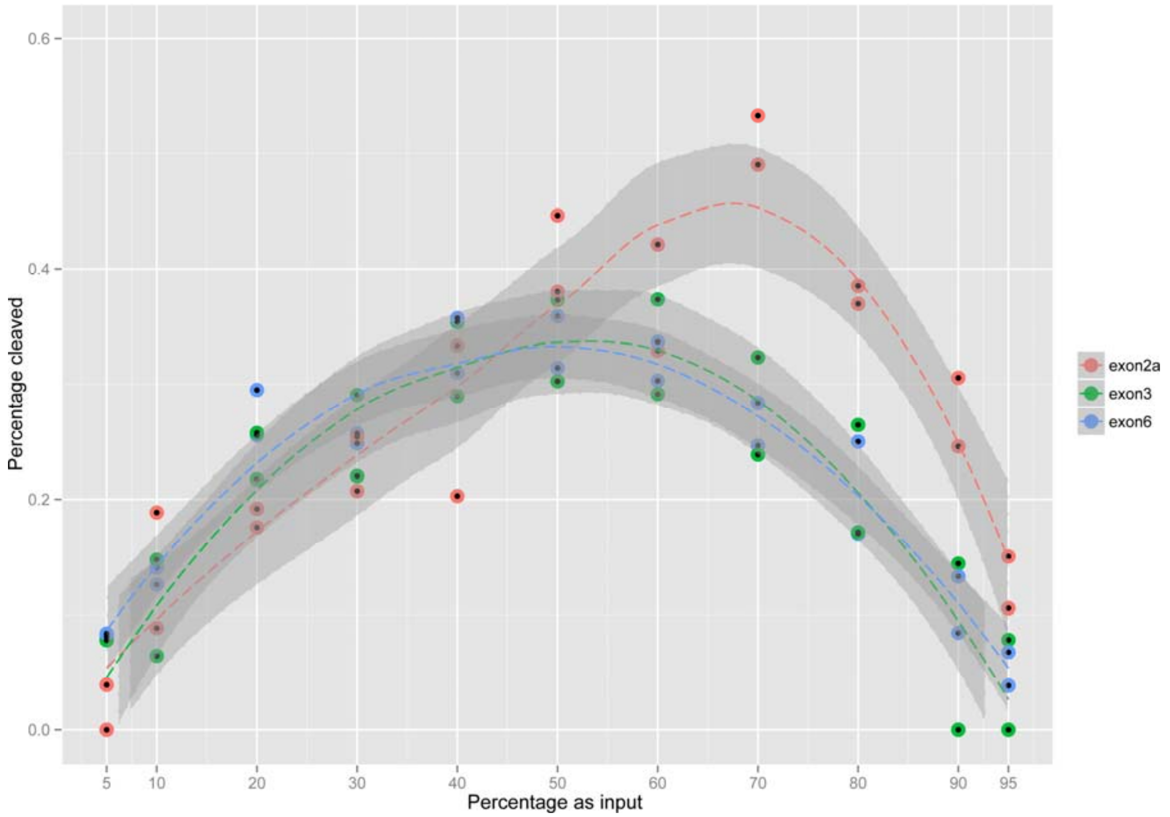

Figure S2 This graph shows the fraction of cleaved products from all products (y axis) in a mixture composed of various quantities of deletion mutants of exon 2a, 3 and 6 in a population of mutant and wild-type DNA molecules (x axis). Both T7E1 and Surveyor digestion products are shown. The grey intervals correspond to the variation and the dotted line corresponds to a loess regression model fitting.

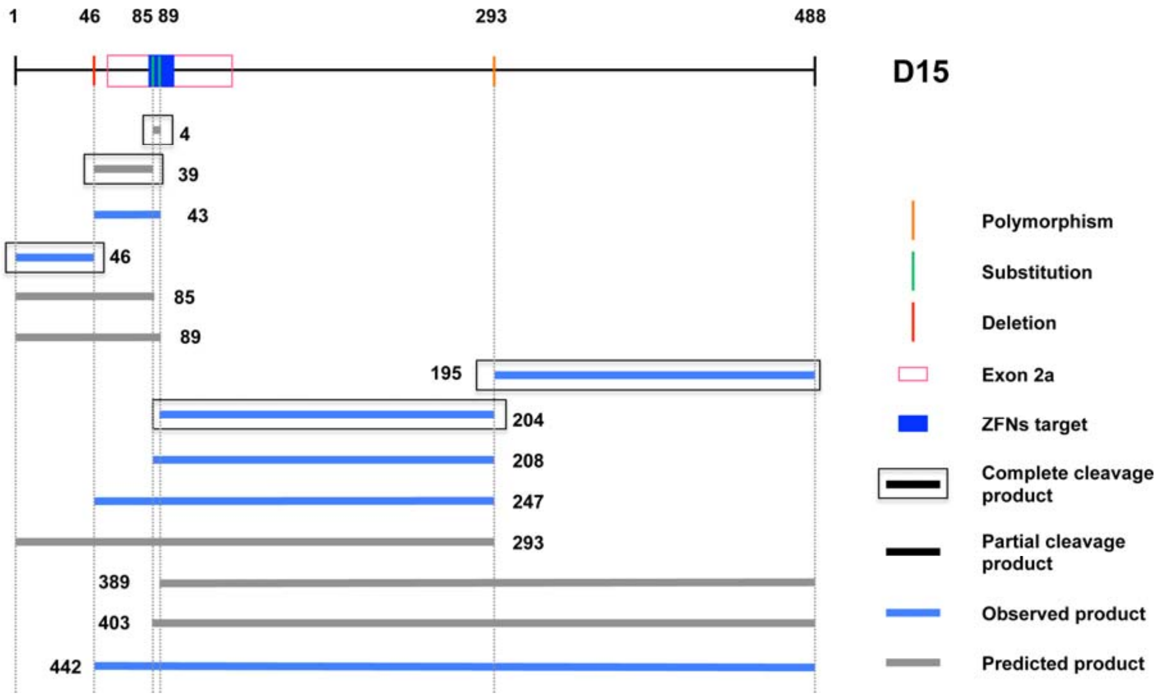

Figure S3 Predicted cleavage map of all mismatches between D15 and wild-type *smn* exon 2a alleles.

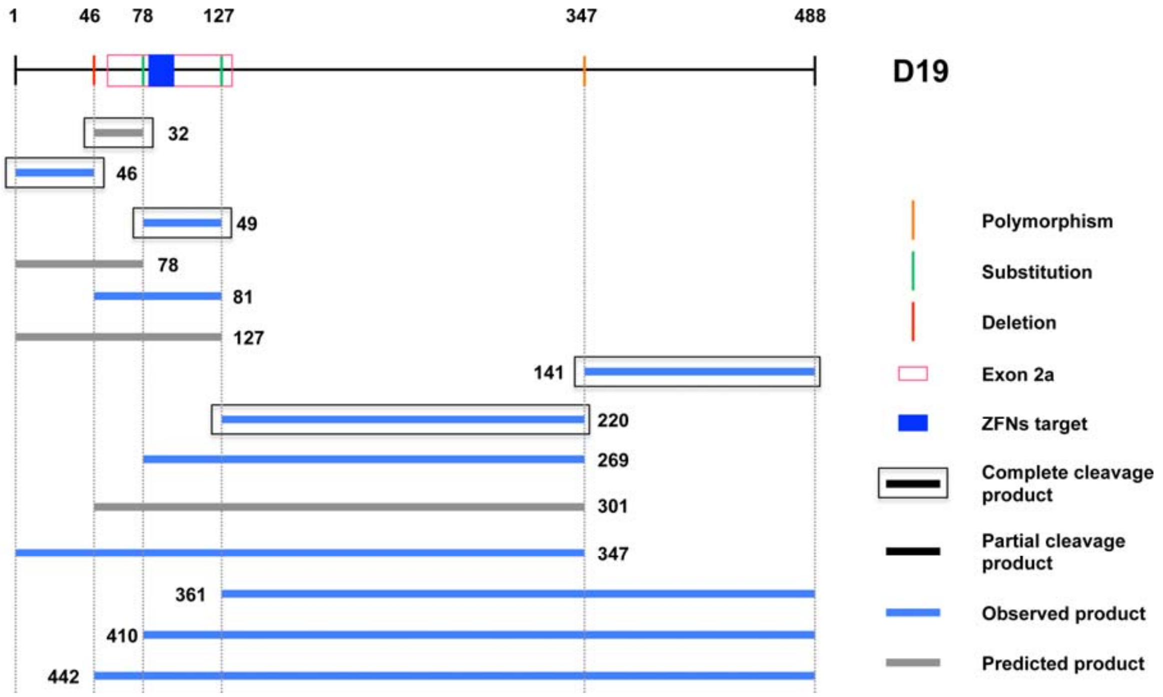

Figure S4 Predicted cleavage map of all mismatches between D19 and wild-type *smn* exon 2a alleles.

Table S1 Sequence of primers used in this work

smn2 gene	Primer sequences 5'-3'		
	Forward	Reverse	Ta (°C)
exon2a	**TCTCCAACTCCTTGTGTTTGTG**	**TATTCCAACACAGGGGCCAC**	58
exon3	**TGCCAAACGAATGAATGTTG**	**CCTTAGCACCTAGGCAGTGG**	50
exon6	**TTCACATTCAAGAAGTCGATAGAA**	**GAATTGATAGATGCAGATCAACC**	50
Constructions of mutants	Primer sequences 5'-3'		
	Forward	Reverse	Ta (°C)
20del_exon2a	**GATACAGCTCTCATT**TCTTCTTTTAAGGTAAGAAT	**TACCTTAAAAGAAGA**AATGAGAGCTGTATCGTCCC	58
20del_exon3	**TGGCTGACCTGCGCT**GAAAGTGACCAGAGGGAGCA	**CCTCTGGTCACTTTC**AGCGCAGGTCAGCCAAACTT	50
20del_exon6	**TCCTCCAATGAGCCC**AGGCATTGGGCAGTATGCTG	**TACTGCCCAATGCCT**GGGCTCATTGGAGGAGGTGG	50

Table S2 List of fragments obtained after T7E1 or Surveyor assays on D15 and D19 clones.

Sample	Observed Size (bp)*	Predicted size of matching fragment (bp)		
		Complete digestion	Partial digestion	Uncleaved
D15 T7E1	23			
**D15 T7E1**	**47**	**46**	**43**	
**D15 T7E1**	**210**	**195, 204**	**208**	
**D15 T7E1**	**250**		**247**	
D15 T7E1	480			
D15 T7E1	*516*			488
D15 Surveyor	51	46		
D15 Surveyor	55			
D15 Surveyor	197	195		
**D15 Surveyor**	**212**	**204**	**208**	
**D15 Surveyor**	**251**		**247**	
**D15 Surveyor**	**426**		**442**	
D15 Surveyor	*516*			488
D19 T7E1	53	46, 49		
**D19 T7E1**	**91**		**81**	
D19 T7E1	223	220		
**D19 T7E1**	**257**		**269**	
**D19 T7E1**	**357**		**347**	
**D19 T7E1**	**373**		**361**	
**D19 T7E1**	**388**		**410**	
**D19 T7E1**	**471**		**442**	
D19 T7E1	*518*			488
D19 Surveyor	143	141		
**D19 Surveyor**	**253**		**269**	
**D19 Surveyor**	**373**		**361**	
**D19 Surveyor**	**438**		**442**	
D19 Surveyor	472			
D19 Surveyor	*513*			488

ND: Non determined
* The observed size is according to the Agilent fragment analysis Expert software report. Sizing accuracy coefficient of variation is +/- 10%, and increases with size.
