# Supplementary material for: Comparison of T7E1 and Surveyor Mismatch Cleavage Assays to Detect Mutations Triggered by Engineered Nucleases
Source: G3 (Bethesda). 2015 Jan 7;5(3):407–15. doi: 10.1534/g3.114.015834 (PMC4349094; doi:10.1534/g3.114.015834)
Supplement: Supporting Information [file supp_g3.114.015834_FigureS1.pdf]

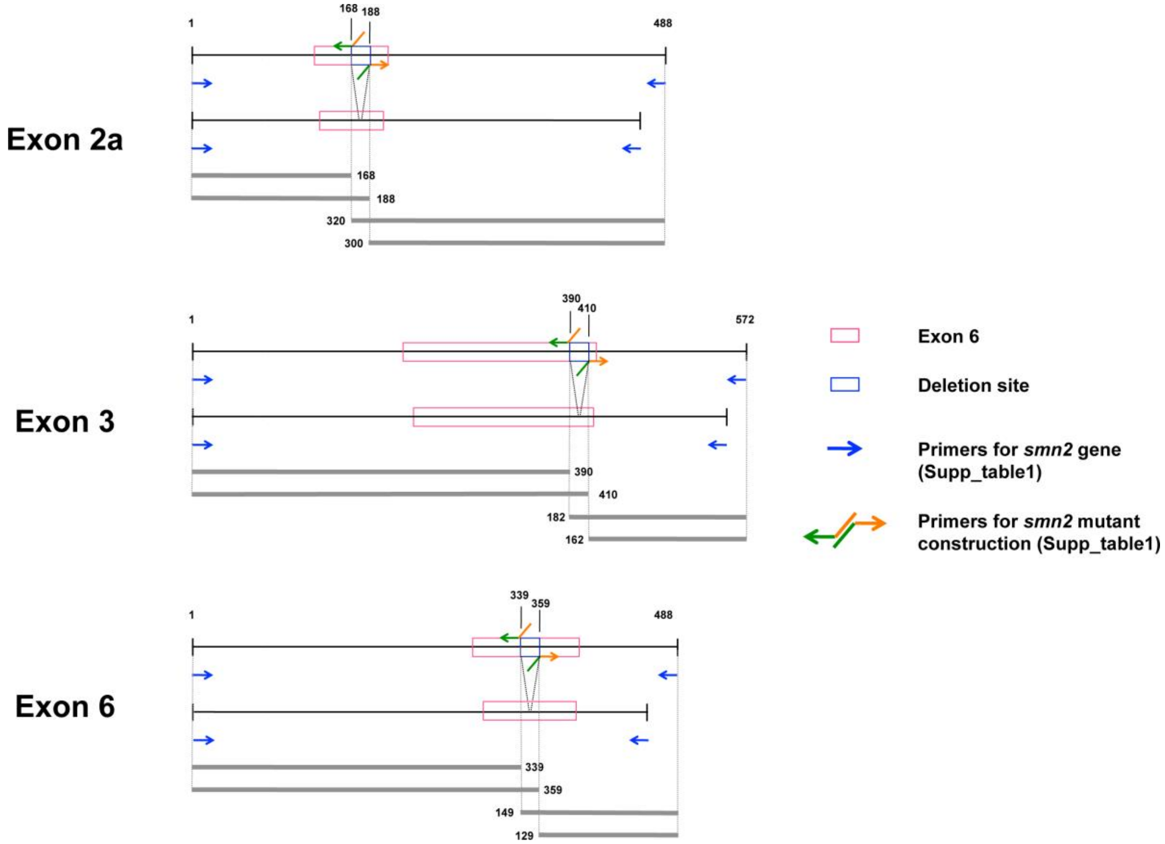

Figure S1 Structure of the PCR products corresponding to *smn* exon 2a, exon 3 and exon 6. Both the wild-type and constructed mutant forms are depicted.
