# Supplementary material for: Comparison of T7E1 and Surveyor Mismatch Cleavage Assays to Detect Mutations Triggered by Engineered Nucleases
Source: G3 (Bethesda). 2015 Jan 7;5(3):407–15. doi: 10.1534/g3.114.015834 (PMC4349094; doi:10.1534/g3.114.015834)
Supplement: Supporting Information [file supp_g3.114.015834_FigureS2.pdf]

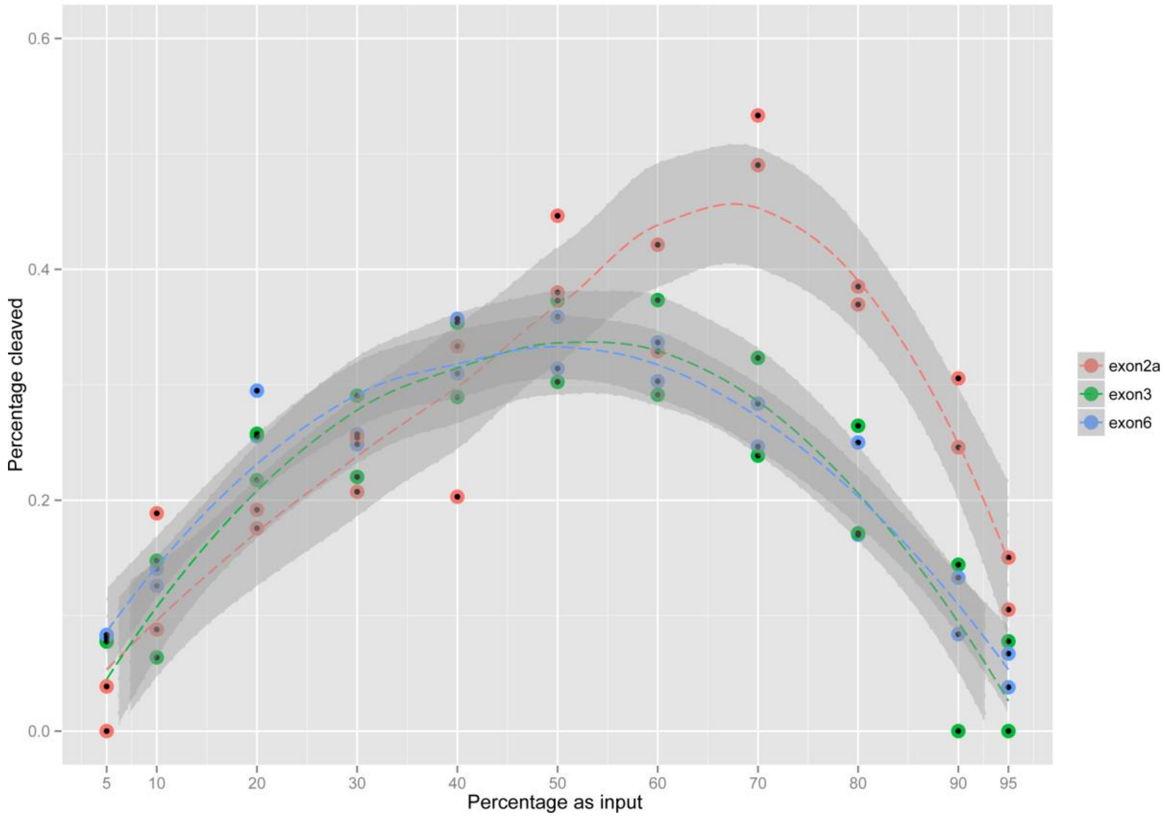

Figure S2 This graph shows the fraction of cleaved products from all products (y axis) in a mixture composed of various quantities of deletion mutants of exon 2a, 3 and 6 in a population of mutant and wild-type DNA molecules (x axis). Both T7E1 and Surveyor digestion products are shown. The grey intervals correspond to the variation and the dotted line corresponds to a loess regression model fitting.
