# Supplementary figures and images for: Comparison of T7E1 and Surveyor Mismatch Cleavage Assays to Detect Mutations Triggered by Engineered Nucleases
Source: G3 (Bethesda). 2015 Jan 7;5(3):407–15. doi: 10.1534/g3.114.015834 (PMC4349094; doi:10.1534/g3.114.015834)

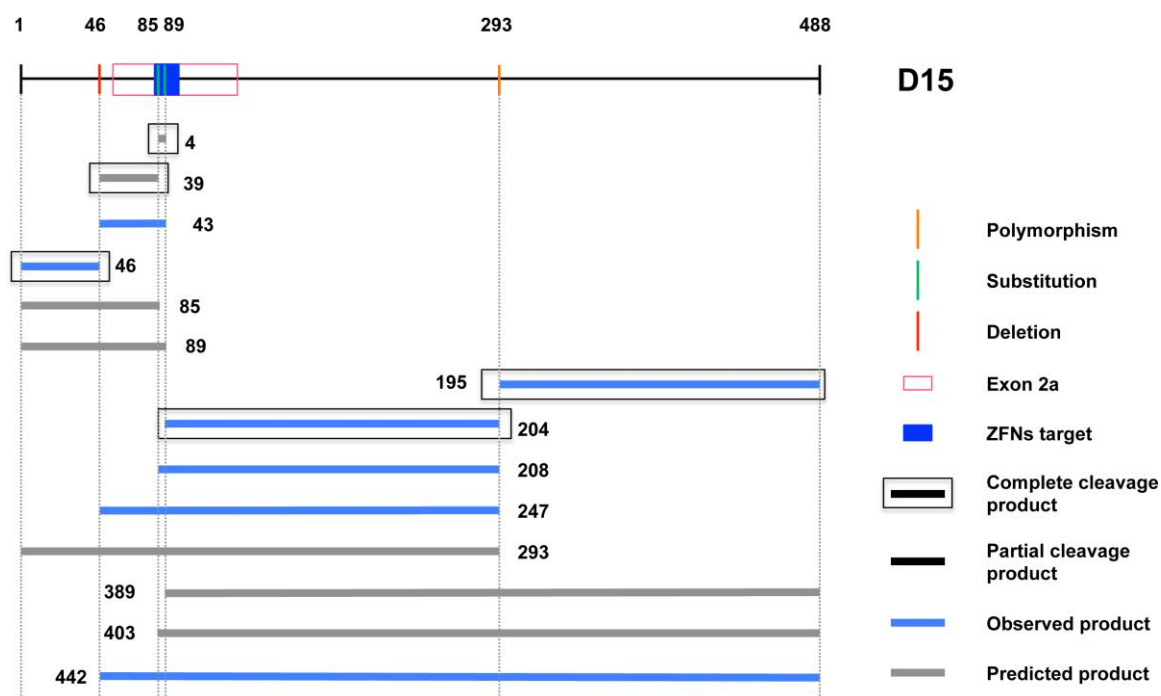

**Figure S3** Predicted cleavage map of all mismatches between D15 and wild-type *smn* exon 2a alleles.

Supplement: Supporting Information [file supp_g3.114.015834_FigureS3.pdf]

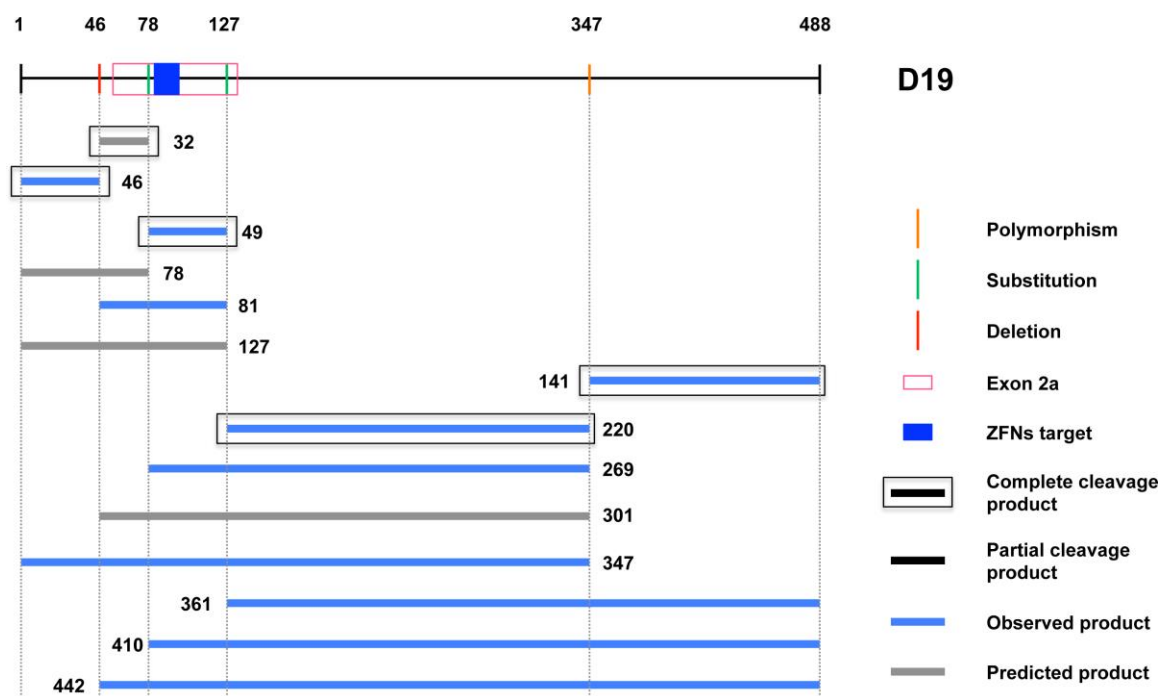

**Figure S4** Predicted cleavage map of all mismatches between D19 and wild-type *smn* exon 2a alleles.

Supplement: Supporting Information [file supp_g3.114.015834_FigureS4.pdf]
