# Supplementary material for: Comparison of T7E1 and Surveyor Mismatch Cleavage Assays to Detect Mutations Triggered by Engineered Nucleases
Source: G3 (Bethesda). 2015 Jan 7;5(3):407–15. doi: 10.1534/g3.114.015834 (PMC4349094; doi:10.1534/g3.114.015834)
Supplement: Supporting Information [file supp_g3.114.015834_TableS1.pdf]

Table S1 Sequence of primers used in this work

smn2 gene	Primer sequences 5'-3'		
	Forward	Reverse	Ta (°C)
exon2a	**TCTCCAACTCCTTGTGTTTGTG**	**TATTCCAACACAGGGGCCAC**	58
exon3	**TGCCAAACGAATGAATGTTG**	**CCTTAGCACCTAGGCAGTGG**	50
exon6	**TTCACATTCAAGAAGTCGATAGAA**	**GAATTGATAGATGCAGATCAACC**	50
Constructions of mutants	Primer sequences 5'-3'		
	Forward	Reverse	Ta (°C)
20del_exon2a	**GATACAGCTCTCATT**TCTTCTTTTAAGGTAAGAAT	**TACCTTAAAAGAAGA**AATGAGAGCTGTATCGTCCC	58
20del_exon3	**TGGCTGACCTGCGCT**GAAAGTGACCAGAGGGAGCA	**CCTCTGGTCACTTTC**AGCGCAGGTCAGCCAAACTT	50
20del_exon6	**TCCTCCAATGAGCCC**AGGCATTGGGCAGTATGCTG	**TACTGCCCAATGCCT**GGGCTCATTGGAGGAGGTGG	50
