# Supplementary material for: Comparison of T7E1 and Surveyor Mismatch Cleavage Assays to Detect Mutations Triggered by Engineered Nucleases
Source: G3 (Bethesda). 2015 Jan 7;5(3):407–15. doi: 10.1534/g3.114.015834 (PMC4349094; doi:10.1534/g3.114.015834)
Supplement: Supporting Information [file supp_g3.114.015834_TableS2.pdf]

Table S2 List of fragments obtained after T7E1 or Surveyor assays on D15 and D19 clones.

Sample	Observed Size (bp)*	Predicted size of matching fragment (bp)		
		Complete digestion	Partial digestion	Uncleaved
D15 T7E1	23			
**D15 T7E1**	**47**	**46**	**43**	
**D15 T7E1**	**210**	**195, 204**	**208**	
**D15 T7E1**	**250**		**247**	
D15 T7E1	480			
D15 T7E1	*516*			488
D15 Surveyor	51	46		
D15 Surveyor	55			
D15 Surveyor	197	195		
**D15 Surveyor**	**212**	**204**	**208**	
**D15 Surveyor**	**251**		**247**	
**D15 Surveyor**	**426**		**442**	
D15 Surveyor	*516*			488
D19 T7E1	53	46, 49		
**D19 T7E1**	**91**		**81**	
D19 T7E1	223	220		
**D19 T7E1**	**257**		**269**	
**D19 T7E1**	**357**		**347**	
**D19 T7E1**	**373**		**361**	
**D19 T7E1**	**388**		**410**	
**D19 T7E1**	**471**		**442**	
D19 T7E1	*518*			488
D19 Surveyor	143	141		
**D19 Surveyor**	**253**		**269**	
**D19 Surveyor**	**373**		**361**	
**D19 Surveyor**	**438**		**442**	
D19 Surveyor	472			
D19 Surveyor	*513*			488

ND: Non determined
* The observed size is according to the Agilent fragment analysis Expert software report. Sizing accuracy coefficient of variation is +/- 10%, and increases with size.
